# Worldwide research trends of pregnancy hypertension in epigenetics field

**DOI:** 10.3389/fpubh.2025.1506992

**Published:** 2025-02-18

**Authors:** Didi Yuan, Yangqing Huang, Hanqi Liu, Haotian Tang, Junwen Liu

**Affiliations:** Department of Histology and Embryology, Xiangya School of Medicine, Central South University, Changsha, Hunan, China

**Keywords:** hypertension in pregnancy (HIP), epigenetic, Citespace, VOSviewer, preeclampsia (PE)

## Abstract

**Introduction:**

Hypertension in pregnancy (HIP) poses significant health risks for both mothers and infants. Development of HIP is influenced by genetic and environmental factors, with epigenetic modifications partially explaining underlying mechanisms. Bibliometric tools aid researchers in quickly gaining insights into field dynamics and trends.

**Methods:**

In this investigation, we conducted a search for relevant publications in the Web of Science Core Collection database using specific keywords. We employed Citespace and WOSviewer software for analysis of interconnections and co-occurrence of information across publications, countries, authors, institutions, keywords and cited literature. Ultimately, we identified 4,316 research papers on hypertension in pregnancy within the epigenetics domain (HIPE).

**Results:**

Our analysis revealed that China had the highest number of publications (*n* = 1,353, 31.35%), while the University of Melbourne was the most prolific institution (*n* = 107, 2.48%). Among author analysis, Tong S emerged as highly productive (*n* = 41, 0.95%). Preeclampsia (PE) emerged as being extensively studied among various types of HIP. High-frequency keywords associated with HIP mechanisms included oxidative stress, proliferation, apoptosis and invasion. Regarding epigenetics-related terms, DNA methylation, mRNA and ncRNA exhibited distinct heat burst periods. The number of HIPE papers demonstrated an upward trend observed through three stages of growth.

**Discussion:**

Our bibliometric-based study provides novel insights into current research progress on HIP from an epigenetic perspective, serving as a source of new ideas and inspiration for future investigations of HIP diseases.

## 1 Introduction

Epigenetics is a sub-discipline of genetics that investigates heritable changes in gene expression without alterations in the nucleotide sequence. In 1942, Conrad Waddington initially proposed the concept of “epigenetic inheritance”: the association between genotype and phenotype (1). Recently, key mechanisms of epigenetic inheritance encompass gene imprinting, DNA methylation, histone modification, non-coding RNA (ncRNA) and N-6-methyladenosine (m6A) modification, which are intricately linked to our environmental milieu. Each organism possesses a distinct genome and epigenome that can be influenced by the environment to activate or suppress gene expression while preserving the DNA sequence. The reversible nature of epigenetic inheritance implies potential reversibility of disease phenotypes, thereby offering novel avenues and insights for disease treatment. Currently, extensive research on epigenetics has been conducted within oncology (2), cardiovascular (3) and immunity (4) fields.

The incidence of HIP affects ~10% of pregnant women worldwide and is clinically characterized by hypertension, edema, proteinuria, convulsions, coma and organ failure (5). HIP can be categorized as chronic (diagnosed before pregnancy or before 20 weeks of gestation) or *de novo* (preeclampsia or gestational hypertension) (6). Current knowledge indicates that there is no effective treatment available for HIP, nor is there an optimal early clinical biomarker to accurately predict its onset (7). Epigenetics links environmental and genetic factors. Studies increasingly demonstrate epigenetic regulation's significant role in HIP development (8, 9). Given the growing interest in exploring the epigenetics-HIP link, it becomes necessary to systematically summarize relevant research.

Bibliometrics is a discipline that focuses on studying the literature system and the characteristics of bibliometrics. It employs mathematical, statistical, and other measurement research methods to analyze the distribution structure, quantitative relationship, changing patterns and quantitative management of literature and intelligence. Additionally, it aims to explore specific structures, characteristics and laws in science and technology (10). This research method enables researchers to quickly comprehend the study's content and trends, as well as identify changes in those trends.

Through the formulation of a comprehensive search strategy, we systematically retrieved an extensive collection of publications pertaining to HIPE from the Web of Science Core Collection (WOSCC) database. Utilizing advanced visualization tools like Citespace and VOSviewer software, we conducted both quantitative and qualitative analyses on various aspects including publications, countries, institutions, disciplines, authors, keywords and co-citations. This approach enables researchers to swiftly grasp the current landscape of HIPE research while providing a valuable reference for future research.

## 2 Materials and methods

### 2.1 Data sources and retrieval strategy

To ensure data quality and comprehensiveness, we sourced study data from Web of Science Core Databases (http://www.webofknowledge.com/). Prior to retrieval, we established inclusion and exclusion criteria for selecting study data. Inclusion criteria included: (1) data obtained from the Web of Science database; (2) data epigenetically related to HIP; (3) data cut-off date of 1 November 2023; (4) literature type limited to “articles” or “reviews”; (5) English-language literature only. Exclusion criteria included: (1) data not related to HIP and epigenetics; (2) literature types of conference proceedings, books, letters, etc.; (3) languages other than English.

The comprehensive search was conducted in the Web of Science Core Collection (WOSCC) database using the keywords “hypertension in pregnancy” and “epigenetic.” To ensure maximum data retrieval, we expanded and refined our search terms. The final search query included the following: (TS = pregnancy hypertension OR gestational hypertension OR hypertension of pregnancy OR hypertension in pregnancy OR pregnancy-induced hypertension OR pregnancy hypertension syndrome OR PIH OR HIP OR eclampsia OR preeclampsia OR HELLP syndrome OR HELLP) AND TS = (epigenetic OR DNA methylation OR chromatin remodeling OR genomic imprinting OR DNA modification OR nucleosome OR histone modification RNA modification OR RNA methylation OR non-coding RNA OR lncRNA OR mRNA OR tRNA OR rRNA OR miRNA OR piRNA OR siRNA OR miRNA OR snRNA OR snoRNA OR RNA editing OR m6A). The search results were further refined by setting the time period from database inception to 1 November 2023, limiting document type to “ARTICLE” and “REVIEW” and selecting English language publications.

### 2.2 Data collection data and analysis

We exported the 4,316 retrieved papers as complete records and eliminated duplicates to obtain the final dataset. The data were imported into the bibliometric platform (https://bibliometric.com), Citespace VI (Version 6.1 R6; https://Citespace.podia.com) and VOSviewer (Version 1.6.18; https://www.vosviewer.com) for systematic analysis. The bibliometric platform was utilized for inter-country collaboration and annual publication analysis, while VOSviewer conducted cluster analysis of institutions, authors and keywords. Citespace VI was employed for burst detection and timeline analysis.

## 3 Results

### 3.1 Time-trend analysis of publication yields

**Figure 1:**
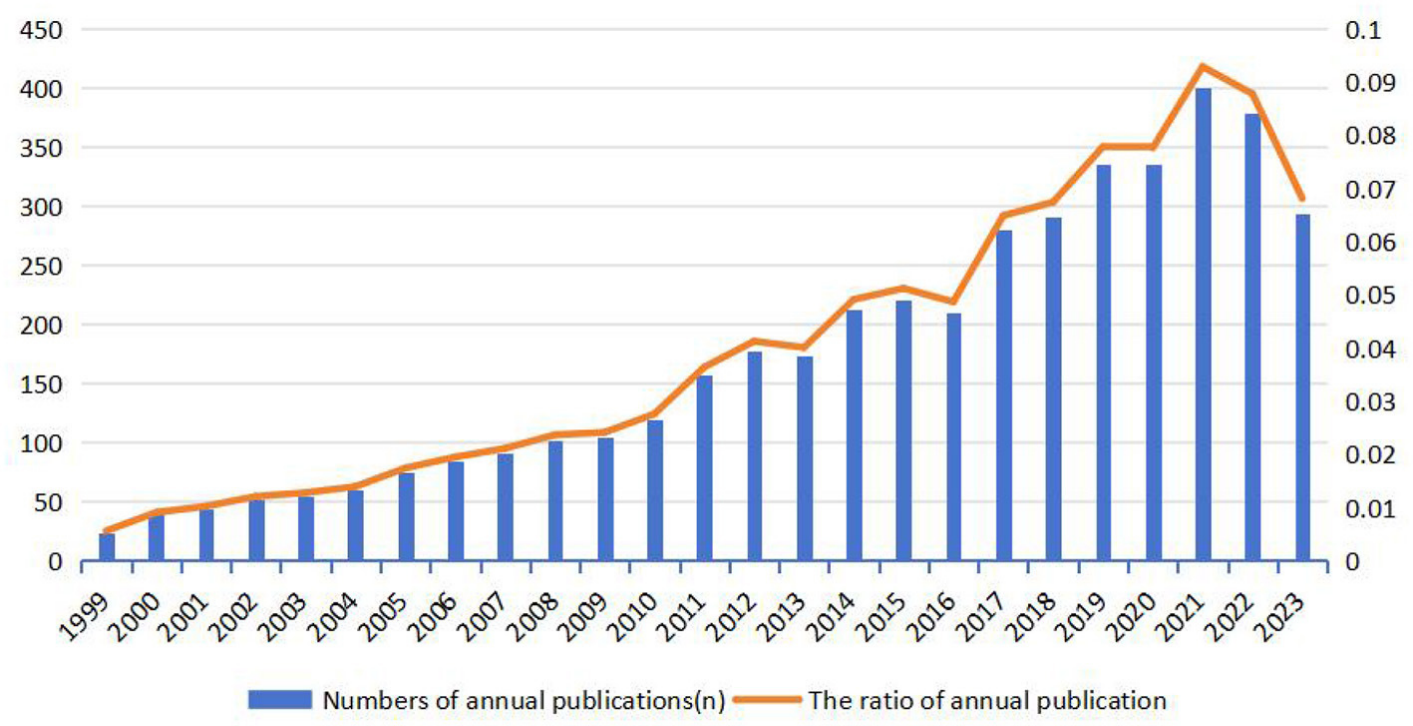
Publication on HIP in the epigenetic field over time.

Chronologically analyzing the 4,316 publications (Figure 1), we identified three distinct phases in the HIPE literature—initial development, rapid expansion and established growth. The earliest relevant research emerged in 1999, with publication volumes remaining below 150 until 2010. During this period, there was a gradual increase in annual publication volume, indicating limited research focus on HIPE and primarily serving as an enlightening stage. Subsequently, from 2011 to 2021, there was a remarkable surge in literature output, reaching its peak of 401 articles in 2021. However, post-2021 witnessed a decline in annual publication volume as the field entered a phase of stable development.

### 3.2 Nation

**Figure 2:**
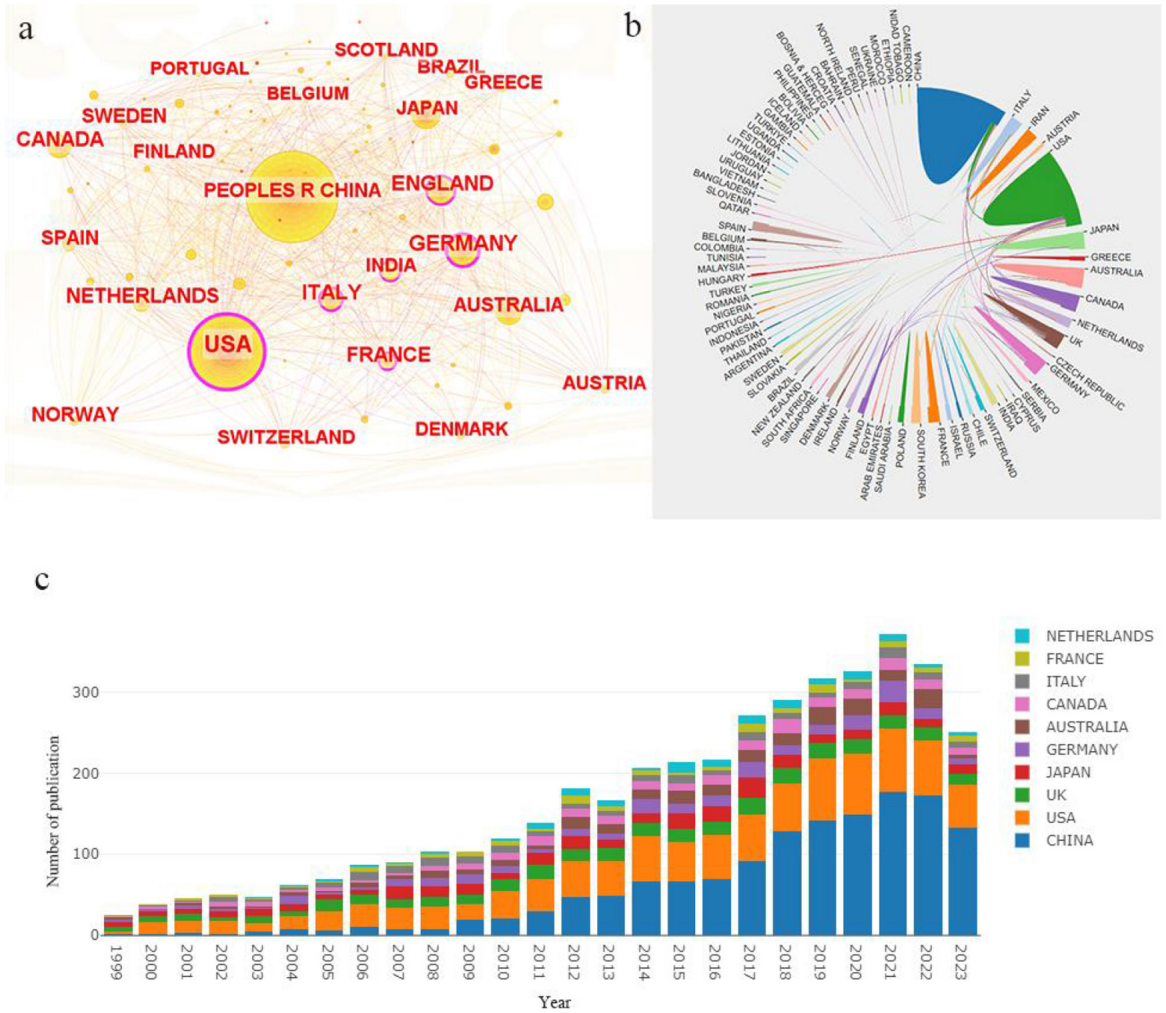
National network visualization analysis in the epigenetics of HIP. **(A)** Country co-occurrence map in Citespace with the purple circle representing centrality. **(B)** Cooperation network among all countries. **(C)** Proportion of articles published by country each year from 1999 to 2023.

According to the statistics, a total of 4,316 literature sources were primarily contributed by 85 countries. The top three countries are China (*n* = 1,353, 31.35%), the United States (*n* = 1,001, 23.19%) and Japan (*n* = 301, 6.97%). Additionally, the countries with the highest centrality values are the United States (0.57), Germany (0.24) and England (0.16) as shown in Table 1. The country centrality typically indicates a country's influence and dominance in a specific discipline or field. Figure 2A visually represents country centrality where the thickness of the purple outer circle corresponds to each country's centrality value. Figure 2B illustrates a mapping of country publication volume versus collaboration, highlighting significantly higher publication volumes for both China and the United States compared to other countries, and revealing that collaboration lines involving the United States are more frequent than those of other nations. Visualizing annual publication volumes for each country (Figure 2C), we observe a rapid growth in China's annual publication volume since 2009 which has subsequently stabilized as being ranked first globally after 2014. The annual publication volume of the United States maintains a slow growth trend while that of other countries such as the United Kingdom, Japan, Germany, Australia and Canada remains stable.

**Table 1:** The top 10 countries by documents.

**Rank**	**Countries/regions**	**Record count**	**Percentage**	**Centrality**
1	PEOPLES R CHINA	1,353	31.35	0.03
2	USA	1,001	23.19	0.57
3	JAPAN	301	6.97	0.04
4	ENGLAND	279	6.46	0.16
5	GERMANY	241	5.58	0.24
6	AUSTRALIA	239	5.54	0.07
7	CANADA	209	4.84	0.05
8	ITALY	166	3.85	0.12
9	FRANCE	121	2.80	0.15
10	NETHERLANDS	121	2.80	0.06

### 3.3 Institution

The study reveals that a total of 183 institutions made contributions to this research endeavor. Table 2 presents the top 10 institutions based on their publication count, along with their centrality, half-life and country affiliation. Centrality reflects the extent of collaboration for each research institution, while half-life indicates its influence. The three leading institutions in terms of publication count are the University of Melbourne (*n* = 94), Nanjing University (*n* = 83) and Shanghai Jiaotong University (*n* = 77). Notably, the University of California (centrality 0.23, half-life 18.5) and Harvard University (centrality 0.35, half-life 15.5) exhibit the highest levels of centrality and half-life respectively, signifying their pivotal role in facilitating institutional collaborations as well as the enduring impact and significance of their publications. These prominent institutions represent China (*n* = 5), United States (*n* = 2), France (*n* = 2) and Australia (*n* = 1). Amongst these top 10 institutions, China possesses the largest representation, however none exhibit high centrality which may be attributed to China's relatively recent engagement in HIPE research and developing collaborative networks.

**Table 2:** The top 10 institution by documents.

**Rank**	**Institution**	**Record count**	**Centrality**	**Country**	**Half-life**
1	University of Melbourne	94	0.19	Australia	8.5
2	Nanjing Medical University	83	0.06	China	7.5
3	Shanghai Jiao Tong University	77	0.06	China	9.5
4	UDICE-French Research Universities	66	0.12	France	9.5
5	Harvard University	62	0.35	USA	15.5
6	Institut National de la Sante et de la Recherche Medicale (Inserm)	59	0.04	France	12.5
7	Fudan University	57	0.08	China	5.5
8	University of California System	56	0.23	USA	18.5
9	Huazhong University of Science & Technology	52	0.03	China	12.5
10	Shandong University	39	0.03	China	3.5

**Figure 3:**
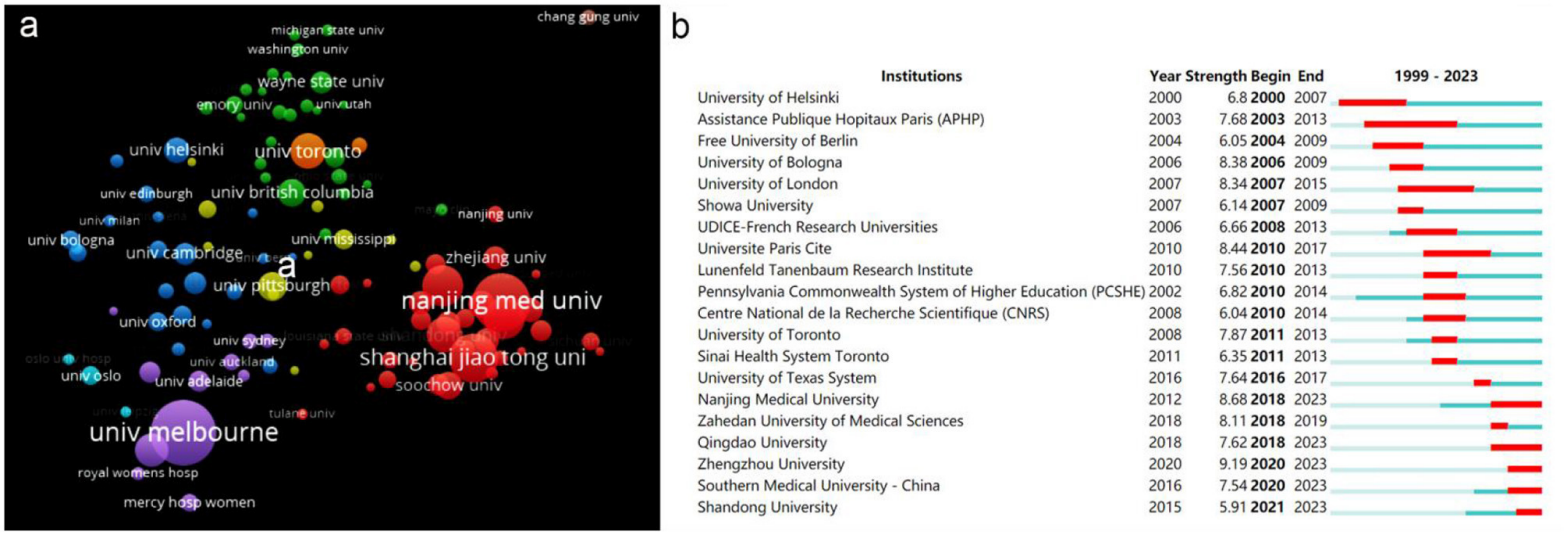
The institution Network visualization analysis in the epigenetic of HIP. **(A)** The cooperation network among all institutions. The size of the circle represents the number of literature and different colors denote different clusters. **(B)** Top 20 institutions with the strongest citation bursts.

Collaboration between institutions was analyzed using VOSviewer. We applied a minimum threshold of 5 agency releases, resulting in inclusion of 110 agencies meeting this criterion. Employing cluster analysis, these institutions were categorized into eight distinct clusters (Figure 3A), each with a unique color code. The largest cluster denoted by the red region is predominantly Chinese institutions such as Nanjing University, Shanghai Jiaotong University and Fudan University exhibiting close collaborative ties. The second largest cluster characterized by a purple hue, revolves around the partnership between the University of Melbourne and Monash University.

To gain deeper insight into the citation profiles of these institutions, this study conducted a comprehensive citation burst analysis using Citespace software (Figure 3B). Our findings reveal that the University of Helsinki has been actively engaged in research within this field for an extended period, evidenced by its sustained and enduring citation impact. Conversely Nanjing University, Qingdao University, Zhengzhou University, Central South University and Shandong University have emerged as noteworthy research institutions with relatively recent citation bursts. It is worth noting that all of these institutions are based in China.

### 3.4 Subject categories

**Figure 4:**
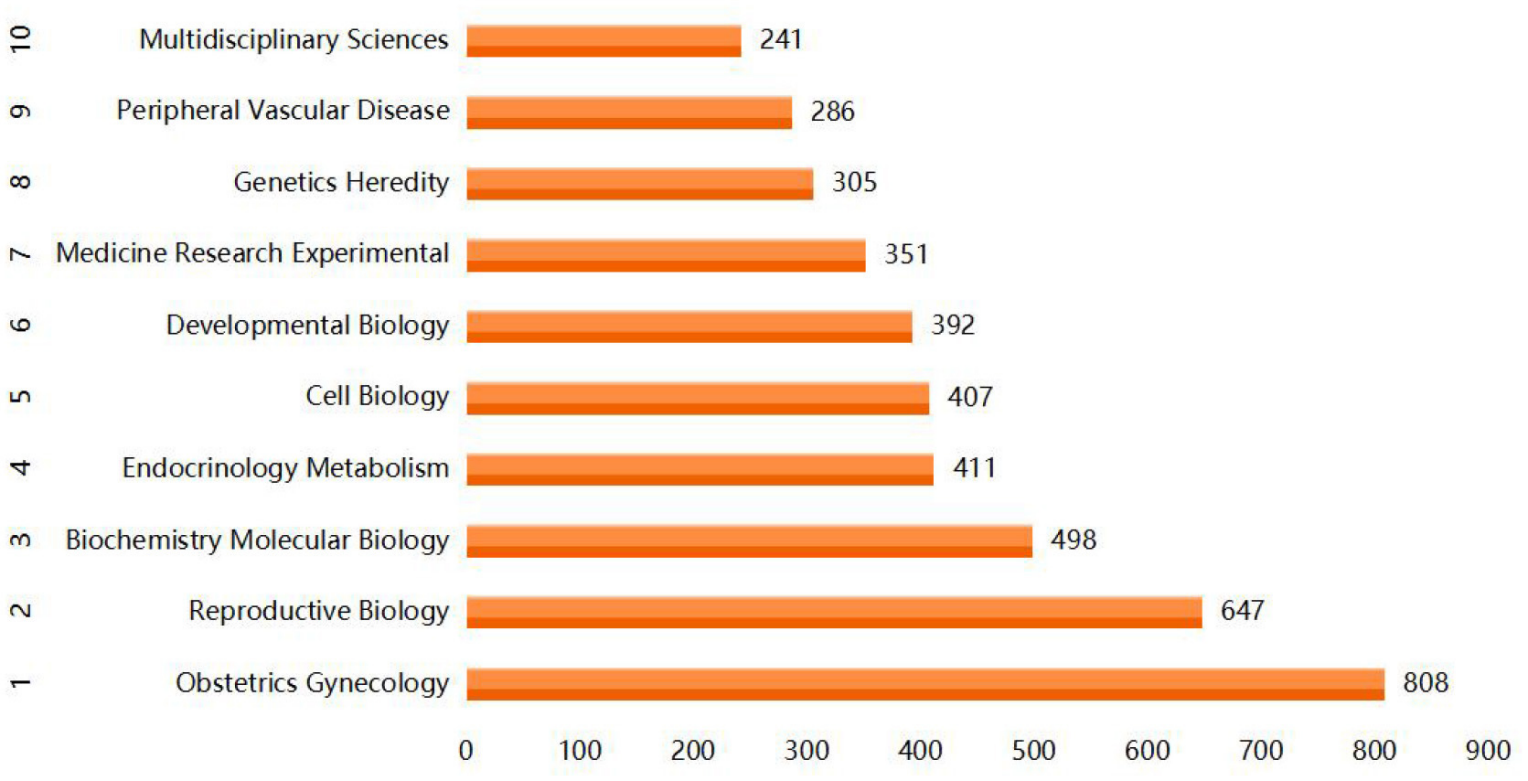
Top 10 web of science categories related to HIPE.

The literature is classified into 254 distinct subject categories by WOS. Analyzing subject classification provides valuable insights into current research focus and interdisciplinary studies. A total of 4,316 articles cover a wide range of disciplines (*n* = 108) (Figure 4). Obstetrics and Gynecology (*n* = 808, 18.70%) emerges as the most prominent discipline with respect to publications, indicating its primary role in current HIPE research. Peripheral vascular disease (*n* = 286, 6.62%) represents an interdisciplinary field closely associated with HIP pathology. Other significant medical foundational disciplines include genetics (*n* = 305, 7.06%), reproductive biology (*n* = 647, 14.97%), chemical molecular biology (*n* = 411, 9.5%), cell biology (*n* = 407, 9.4%), developmental biology (*n* = 392, 9.07%) and medical research experiment (*n* = 351, 8.1%). These disciplines encompass a substantial body of research on HIPE pathological mechanisms.

### 3.5 Authors

We analyzed 4,316 publications and identified that the study involved contributions from 21,881 authors. Table 3 presents the top 10 authors based on their publication output and citation impact. Tong S (*n* = 41, 1.37%), Hannan NJ (*n* = 34, 1.24%) and Kaitu'u-lino TJ (*n* = 32, 1.24%) emerged as the leading contributors in terms of publication output, all affiliated with the University of Melbourne. This finding suggests that the University of Melbourne has established a well-established research team specializing in HIPE research, with Tong S serving as its core member. The number of citations received by an author reflects their influence within the field, hence we observed Robinson Wendy P (*n* = 947), Burton Graham J (*n* = 864) and Karumanchi SA (*n* = 817) from the University of British Columbia, University of Cambridge and Harvard University, respectively, as the top three most cited authors overall. Notably, among them are Tong S, Robinson WP and Sekizawa A who rank among the top ten both in terms of publications and citations—indicating their pivotal role in advancing HIPE research.

**Table 3:** The top 10 authors by documentts and the top 10 co-authors by citations.

**Author**	**Institution**	**Documents**	**Co-author**	**Institution**	**Citations**
Tong, Stephen	Univ Melbourne	41	Robinson, Wendy P	Univ British Columbia	947
Hannan, Natalie J	Univ Melbourne	34	Burton, Graham J	Univ Cambridge	864
Kaitu'u-lino, Tu'uhevaha J	Univ Melbourne	32	karumanchi SA	Harvard Univ	817
Cannon, Ping	Univ Melbourne	22	Vaiman D	Univ Paris	690
Sekizawa, Akihiko	Showa Univ	21	Sekizawa, Akihiko	Showa Univ	661
Robinson, Wendy P	Univ British Columbia	21	Tong, Stephen	Univ Melbourne	650
Xu, Zhice	Soochow Univ	18	Farina A	Univ Bologna	641
Sun, Lizhou	Nanjing Med Univ	17	Romero R	Wayne State Univ	631
Purwosunu, Yuditiya	Univ Indonesia	17	Purwosunu Y	Showa Univ	592
Zhang lubo	Loma Linda Univ	17	Okai T	Showa Univ	572

**Figure 5:**
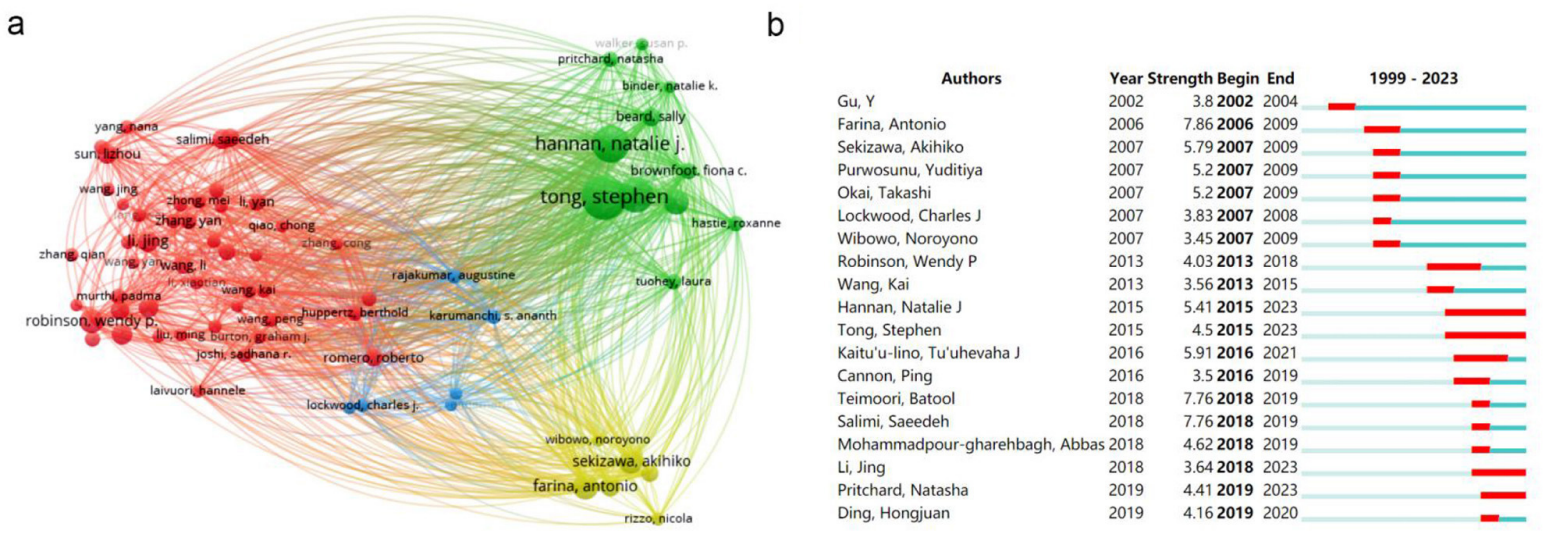
The author network visualization analysis in the epigenetics of HIP. **(A)** Cooperation network among all authors. The size of the circle represents the number of publications and the thickness of the connection represents the degree of association. **(B)** Top 19 authors with the strongest citation bursts.

To further investigate the collaboration among authors, we set a minimum threshold of 10 literature publications in WOSviewer, identifying 74 authors meeting this criterion. Coupling analysis revealed four distinct clusters (Figure 5A), represented by colors red, yellow, blue and green. The largest red cluster centered on Robinson WP, Li Jing and Salimi S, primarily focused on exploring epigenetic mechanisms regulating blood pressure during preeclampsia at both DNA methylation and ncRNA levels (11–13). The green cluster centered on Tong S and Hannan NJ, predominantly investigated placenta-associated mechanisms in blood pressure regulation during preeclampsia (14, 15). The yellow cluster with Sekizawa A and Farina A at its core emphasized studying differential expression patterns of placenta-derived cellular mRNAs within maternal blood samples from preeclampsia women (16, 17).

To further investigate the key authors currently involved in this study, we conducted author citation burst detection using Citespace (Figure 5B). The burst detection reveals the initiation and duration of prominence for each identified term. Gu Y emerges as an early influential figure in this field. Farina A, Sekizawa A, Purwosunu Y, Okai T, Lockwood CJ and Wibowo N are associated with a research outbreak during the period of 2006–2009, suggesting their work represents significant research hotspots during that time frame. Hannan NJ, Tong S, Li J and Pritchard N are authors who have gained momentum after 2015 and continue to be at the forefront of current research on HIPE. Analyzing their findings can provide insights into contemporary research hotspots and frontiers within this domain. Notably, Hannan NJ and Tong S stand out as highly influential authors with enduring impact.

### 3.6 Keywords

Keywords provide a concise representation of a research field, facilitating rapid comprehension of its direction and dynamics. A total of 14,352 keywords were extracted from the topics and abstracts of 4,316 documents. Table 4 presents the top 20 keywords based on their frequency. Analysis of these keywords reveals that preeclampsia is extensively studied within HIP, highlighting its significance in maternal and infant outcomes. Keywords such as oxidative stress, proliferation, apoptosis and invasion are closely associated with the underlying pathomechanism of HIP. Additionally DNA methylation emerges as the most extensively researched regulatory mechanism within HIPE. Notably, Robinson WP's team has made significant contributions to investigating placental DNA methylation differences and their clinical implications (18–20).

**Table 4:** The top 20 keywords by documents and centrality.

**No**.	**Keyword**	**Frequency**	**Centrality**	**No**.	**Keyword**	**Frequency**	**Centrality**
1	Expression	1,104	0.17	11	Apoptosis	229	0.02
2	Preeclampsia	806	0.11	12	Gene	216	0.03
3	Pregnancy	708	0.1	13	Invasion	213	0.02
4	Gene expression	478	0.17	14	Blood pressure	209	0.07
5	DNA methylation	416	0.06	15	Differentiation	209	0.04
6	Hypertension	360	0.06	16	Activation	186	0.06
7	Cells	346	0.07	17	Proliferation	183	0.02
8	Risk	280	0.04	18	Growth	177	0.03
9	Women	279	0.05	19	*in vitro*	176	0.08
10	Oxidative stress	258	0.04	20	Cancer	162	0.05

**Figure 6:**
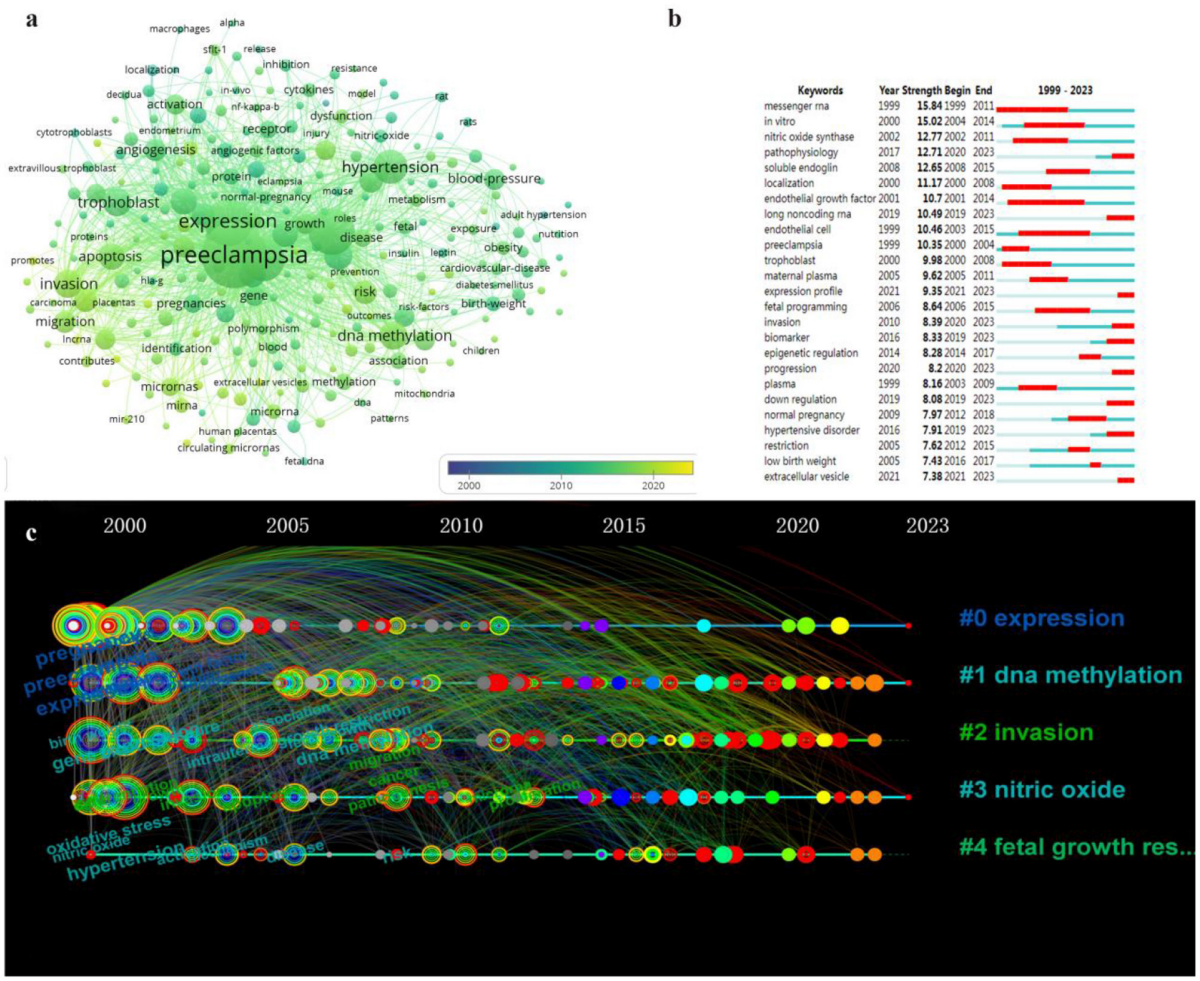
Network visualization analysis of epigenetic keywords related to HIP. **(A)** Co-occurring keywords overlay visualization over time. **(B)** Top 25 keywords with strongest citation bursts related to HIP. **(C)** Time line view of keyword clustering related to HIP.

To gain a deeper understanding of the temporal dynamics of keyword occurrence, we set the minimum frequency threshold for keyword occurrence to 20 in VOSviewer. Consequently, a total of 257 keywords met this criterion (Figure 6A). By examining the time-viewable view, it became evident that these keywords were predominantly concentrated within the period spanning from 2010 to 2020, indicating a peak in research on HIPE during this timeframe. Additionally, employing Citespace allowed us to identify keyword bursts (Figure 6B). Notably, mRNA was investigated as early as 1999 and remained an active area of study until 2011. Conversely, recent years witnessed emerging keywords such as lncRNA, expression profiling, down-regulation and extracellular membrane vesicles with heightened levels of activity. Based on these findings, we postulate that the epigenetics research hotspot pertaining to HIP has shifted from mRNA toward lncRNA.

To further refine the keywords, this study conducted a comprehensive analysis of the keyword clusters using Citespace and generated a timeline graph based on their chronological occurrence (Figure 6C). Each node represents a specific keyword, positioned along the timeline to indicate its earliest appearance, with size corresponding to frequency. Connecting lines between nodes represent link strength. Nodes are color-coded in a gradient from gray to red, with shades closer to gray indicating earlier occurrences and those closer to red representing more recent times. Cluster #0 (expression) predominantly exhibits nodes toward the left side of the timeline, suggesting that expression-related keywords emerged earlier and certain keywords within this cluster remain highly relevant today as indicated by their red outer rings. In contrast, clusters #1 (DNA methylation), #2 (invasion), #3 (nitric oxide) and #4 (fetal growth restriction) display scattered distribution across the timeline, signifying ongoing emergence of new keywords within these clusters.

### 3.7 Citation analysis

**Figure 7:**
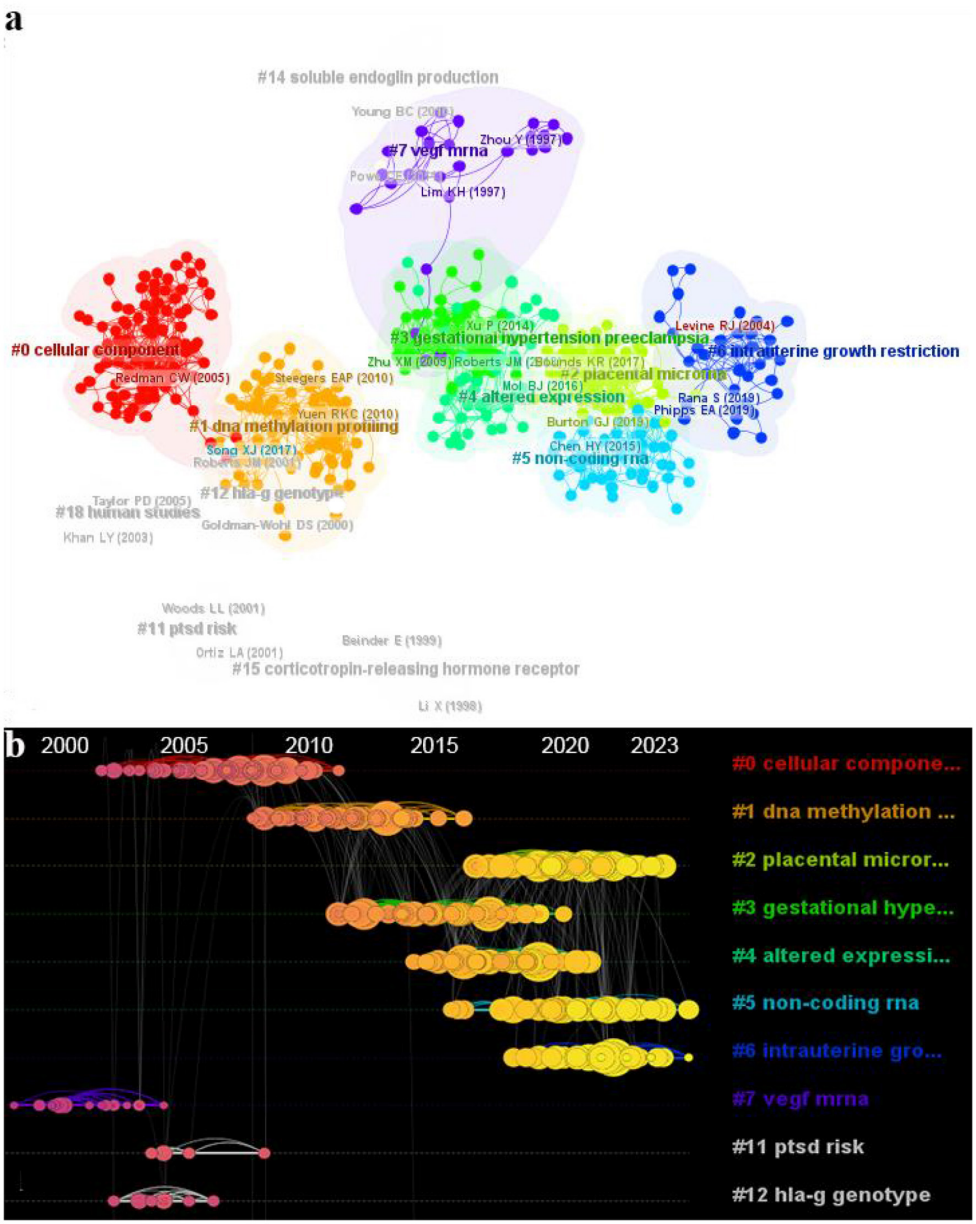
The reference network visualization analysis in the epigenetics of HIP. **(A)** Clusters of references with Citespace. **(B)** Timeline view of co-cited literature related to epigenetic studies of HIP.

We imported 4,316 documents into VOSviewer for analysis and identified a total of 135,018 cited references. To facilitate examination of interrelationships among these references, we employed Citespace for co-citation clustering analysis. Noun terms extracted from titles were utilized to label resulting clusters, generating a total of 18 distinct clusters (Figure 7A). Notably, clustering parameters with Q values > 0.3 indicate significant association structures within the clusters, while S values > 0.7 suggest high confidence in clustering outcomes. The generated clusters exhibited a Q value of 0.7219 and an average S value of 0.8975, affirming their structural stability and remarkable reliability.

Table 5 illustrates the top 10 clusters in terms of size, with the largest cluster (#0) consisting of 92 members and an S-value of 0.917. The LLR algorithm extracted labels related to cellular components, the LSI algorithm extracted placental expression and the MI algorithm labeled metamyelocytes (0.45). These algorithms provided by Citespace were used to extract clustering labels from different locations in the sizing literature. We utilized the LLR algorithm's extracted labels as they emphasize research characteristics of each cluster for nomenclature purposes. Recurring labels such as placenta, DNA methylation, preeclampsia, trophoblasts, endothelial cells and RNA suggest a close association between HIPE and these topics.

**Table 5:** Summary of the largest 10 clusters.

**Cluster ID**	**Size**	**Silhouette**	**Label (LSI)**	**Label (LLR)**	**Label (MI)**	**Average year**
0	92	0.917	Placental expression	Cellular component (443.56, 1.0E-4)	Decidual cell (0.45)	2003
1	86	0.858	Human placenta	DNA methylation profiling (412.16, 1.0E-4)	Tissue-specific inhibition (1.26)	2008
2	60	0.887	Pregnancy complication	Placental microrna (367.03, 1.0E-4)	Tissue-specific inhibition (1.37)	2017
3	59	0.884	Preeclamptic placenta	Gestational hypertension preeclampsia (337.8, 1.0E-4)	7i-induced atg4b suppression (0.74)	2012
4	50	0.859	Severe preeclampsia	Altered expression (330.4, 1.0E-4)	Human decidua (2.47)	2014
5	44	0.839	Trophoblast cell	Non-coding rna (605.85, 1.0E-4)	Cell-derived extracellular vesicle (0.63)	2017
6	36	0.956	Intrauterine growth restriction	Intrauterine growth restriction (302.32, 1.0E-4)	Tissue-specific inhibition (1.06)	2018
7	26	0.998	1 expression	Vegf mrna (115.53, 1.0E-4)	Abnormal fetal growth (0.01)	1997
11	8	0.994	Ptsd risk	Ptsd risk (49.08, 1.0E-4)	Human placenta (0.02)	2001
12	8	0.99	Hla-g genotype	Hla-g genotype (99.67, 1.0E-4)	Human placenta (0.02)	2001

We conducted a temporal analysis of the top 10 clusters in Citespace (Figure 7B). The node size in the graph represents reference frequency, with darker nodes indicating earlier time periods and brighter nodes indicating more recent ones. Our findings reveal that clusters #7 (VEGF mRNA), #11 (PTSD risk) and #12 (hla-g genotype) were more frequently cited before 2010, while clusters #2 (placental microRNA), #5 (non-coding RNA) and #6 (intrauterine growth restriction) gained prominence in the literature after 2010. Cluster #1 (DNA methylation) lies between these two time periods. These temporal shifts in clustering patterns suggest a research focus transition within the epigenetic field of HIP from mRNA to DNA methylation to ncRNA.

## 4 Discussion

The field of epigenetic research conducted by HIP has yielded significant advancements since 1999, yet there is a dearth of literature examining this subject from a bibliometric perspective. In this study, we systematically analyze 4,316 publications employing two econometric research tools: Citespace and VOSviewer.

The field of HIPE has experienced continuous growth, particularly from 2011 to 2023, during which there was a significant increase in published literature leading to a peak. In terms of country analysis, China contributed the most to the number of literatures, however the United States exhibited the highest centrality. This suggests that HIPE research remains predominantly centered in the United States, while Chinese research started relatively late and is yet to establish extensive collaborative networks. The University of California and Harvard University exhibited both high centrality and longevity, indicating that publications from these institutions had substantial impact and durability. Notably, Tong S played a central role within an exceptionally sophisticated HIPE research team at the University of Melbourne. China boasts numerous emerging institutions such as Nanjing University, Qingdao University, Zhengzhou University, Central South University, Shandong University etc. whose delayed citation but high citation rate signifies their growing influence. Tong S, Robinson WP and Sekizawa A rank among the top 10 authors in terms of publications and citations within HIPE research circles, underscoring their pivotal roles in shaping HIPE centers and significantly contributing to its development.

In the keyword analysis, PE exhibits a high frequency of occurrence as one of the classifications for HIP, indicating its predominant nature in HIP research. PE affects 5%−8% of pregnancies and is clinically characterized by new-onset hypertension, proteinuria, or impaired end-organ function after 20 weeks of gestation (21). Based on the time of onset, PE can be categorized as Early Onset PE (EOPE, < 34 weeks) and Later Onset PE (LOPE, >34 weeks). Studies have demonstrated that EOPE presents more significant epigenetic alterations possibly due to its early onset and sufficient time for epigenetic molecules to exert an impact (8).

“Cell” ranks as the 7th most prominent keyword. In the analysis of keyword bursts, the terms “endothelial cells” and “trophoblasts” exhibited sustained high levels of interest throughout the study of HIP, indicating a significant association between these cell types and the occurrence of HIP. Another cell-related keyword that recently emerged is “extracellular vesicles” (Burst Period: 2021–2023). Although “extracellular vesicles” formed a stable cluster in citation analysis, its ranking among keywords was less prominent, suggesting that research on extracellular vesicles in HIP is still in an exploratory phase.

Oxidative stress, proliferation, apoptosis and invasion are high-frequency keywords associated with HIP pathogenesis. Studies have indicated that oxidative stress resulting from trophoblast invasion and remodeling of the maternal spiral artery barrier is a critical mechanism inducing HIP. Dysregulation of trophoblast proliferation and apoptosis significantly affects the invasion ability of trophoblast cells (22). The “oxidative stress” outbreak period emerged between 2002 and 2011 and the “invasion” fever has persisted since 2010. These two keywords form mature keyword clusters, suggesting that oxidative stress and invasion are mature mechanisms leading to HIP. The top-ranking keywords “proliferation” and “apoptosis,” did not form a keyword cluster and were recent outbreaks, indicating that the mechanism affecting trophoblast cell proliferation and apoptosis is a current research hotspot.

The keyword “hypertension” represents a high-frequency keyword described the most prominent clinical symptom of HIP. Abnormal remodeling of uterine spiral arteries and damage to the systemic vascular endothelial system constitute major mechanisms affecting blood pressure in patients with PE. Tong S's team demonstrated that soluble fms-like tyrosine kinase 1 (sFlt-1) was identified as the primary factor influencing blood pressure levels in both circulating blood and placental tissues of PE patients (23, 24).

The keywords associated with epigenetics encompass DNA methylation, messerage RNA (mRNA) and non-coding RNA (ncRNA). Notably, significant temporal variations were observed in the citation analysis of these keywords, aligning with their respective periods of outbreak. mRNA emerged as an early keyword, with VEGF mRNA being frequently mentioned in initial citations. On the other hand, ncRNA emerged as a high-frequency term from 2015 onwards, persisting as such in the cited literature to date. lncRNA a subtype of ncRNA, exprienced a keyword outbreak period spanning from 2019 to 2023. While DNA methylation did not exhibit a distinct outbreak period among keywords, it was prominently cited during the years 2010–2015 according to our citation analysis. Based on these findings, we propose that research focus within the field of epigenetics has shifted from mRNA to DNA methylation and subsequently toward ncRNA.

The best-studied epigenetic modification in PE is DNA methylation, typically within cytosine-phosphoguanine (CpG) dinucleotides (25). Supplementary Table S1 lists abnormal methylation genes identified in the placenta and maternal blood during pregnancy, along with potential pathways through which these genes contribute to PE. ncRNAs are RNA molecules transcribed from the genome that do not encode proteins. From an epigenetic perspective, regulatory ncRNAs are more interesting because they modify other RNAs (26). MicroRNA (miRNA) is a high-frequency word distilled from citations in the last 5 years and lncRNA is a keyword that has recently burst into the spotlight, both of them belong to the category of regulatory ncRNAs. Supplementary Tables S2, S3 list placenta and maternal blood differentially expressed miRNAs and lncRNAs, along with their target genes and potential HIP induction mechanisms.

N-6-methyladenosine (m6A) modification is the most common internal modification in eukaryotic mRNA. In 2012, Rechavi's team first revealed the sequencing method for m6A, which greatly contributed to the development of m6A modifications (27). At present, m6A modification has made important progress in cardiovascular (28) and tumor (29) research. m6A has been studied more and more in the field of HIPE (30–32). In 2022, Zhu Y's team at Zhejiang University first explored the potential function and expression patterns of m6A and its related genes in preeclampsia (33). In a recent review, Sun LZ's team at Nanjing University summarized the relationship between m6A modifications and preeclampsia risk factors such as diabetes, cardiovascular disease, obesity and psychological stress (34). These studies hint at the potential of m6A modification in HIP studies. At present, “m6A” has not formed clustering and outbreak period, and it is not prominent in the keyword ranking. We speculate that m6A modification may be the hot spot and trend of HIPE research in the future.

Fetal growth restriction (FGR) and low birth weight (LBW) are key terms related to fetuses. These two keywords first emerged in 2006 and 2005, respectively. The outbreak period of FGR was from 2006 to 2015, and the outbreak period of LBW was from 2016 to 2017. The popularity of these two keywords continues to this day, especially FGR has formed a mature keyword clustering. This indicates that fetal epigenetic changes in HIP environment have received extensive attention. Numerous studies have demonstrated higher rates of FGR and LBW in women with PE compared to those with normal pregnancies (35). There exists a correlation between PE-related intrauterine exposure, growth restriction, low birth weight and adverse effects on offspring health including heart disease (36), renal dysfunction (37), systemic vascular damage (38), endocrine disorders (39), immune dysfunction (40) and poor neurodevelopmental outcomes (41). This association may be explained by a multifactorial interaction involving genetic background and epigenetic reprogramming, however the precise mechanisms remain unclear. Current research has demonstrated epigenetic associations between HIP and elevated blood pressure in offspring (42–45). Furthermore, the impact of PE on the fetal endothelial transcriptome and endothelial function exhibits sex-specificity, with female offspring being more severely affected than male offspring (46). Increased attention to epigenetic changes and long-term effects in fetuses exposed to HIP helps to explain the characteristics of non-inherited individual differences, but further research findings depend on the development of HIPE.

The biomarker is the keyword for the recent outbreak (Burst Period 2019–2023). In keyword clustering and citation clustering, biomarkers did not form correlation clusters. Biomarkers also did not feature prominently in the keyword rankings. Based on outbreak timing, we speculate that biomarkers are emerging themes. With the advent of HIPE, scientists have sought to identify epigenetic markers that exhibit diagnostic disparities between maternal blood and placenta. Given their heightened specificity compared to DNA methylation differential markers and their stable expression in maternal blood, ncRNAs emerge as superior HIP biomarkers. Sun et al. demonstrated that lncRNA BC030099 (AUC = 0.713) levels in whole blood possess commendable discriminatory potential for distinguishing preeclampsia from healthy pregnancies (47). Additionally, Luo et al. proposed that serum levels of three lncRNAs namely AF085938, G36948 and AK002210 (AUC = 0.7673, 0.7956, and 0.7575 respectively), could serve as promising diagnostic biomarkers for preeclampsia (48). Recent findings from a case-control study revealed significantly elevated expression levels of NR_002187, ENST00000398554, ENST00000586560, TCONS_00008014, ENST00000546789, ENST00000610270, and ENST00000527727 in the blood of affected individuals compared to normal pregnant women, these molecules hold promise for stratifying HIP severity (AUC ranging between 0.6 and 0.7) (49). As specific miRNA patterns in the placenta are dynamically reflected in maternal circulation during pregnancy, several researchers have advocated for the use of miRNAs as diagnostic markers for PE (50, 51). The chromosome 19 microRNA cluster (C19MC) located on an imprinted paternally inherited allele on the human chromosome Chr19q13 locus is one of the largest human miRNA clusters expressed almost exclusively in the placenta. Therefore C19MC was once considered one of the most reliable markers defining early pregnancy trophoblast cells (52). While these epigenetic markers have enabled early diagnosis of PE, their universal application still faces challenges such as the low abundance of circulating lncRNA limits quantification and standardization and the confounding of non-specific miRNAs and exosomes as well as seed sequences shared by miRNAs reduce the sensitivity of miRNAs as diagnostic agents. Additionally sample processing, validated assays and consensus among researchers on PE also affect its utility. More new epigenetic markers need to be further discovered.

## 5 Conclusion

The rapid advancement of research on HIP in the field of epigenetics signifies its extensive research prospects. This study presents a pioneering bibliometric analysis of global research patterns and collaborative networks in HIPE enabling researchers to promptly comprehend the current status of HIPE research. The findings reveal that the United States remains the primary hub for HIP research, while Chinese researchers are striving to bridge this gap. Notably, publications from esteemed institutions such as the University of California and Harvard University exhibit substantial impact and longevity. Moreover, the University of Melbourne has cultivated a highly proficient HIPE research team with Dr. Tong S. serving as its core member. PE is the main research type of HIP. Epigenetic studies have transitioned from focusing on mRNA and DNA methylation to ncRNA, and m6A modification potentially representing the hot spot and trend of future research. According to the keyword timeline analysis, fetal epigenetic effects and biomarkers are emerging research topics after mechanism study. It should be noted that our study may possess certain biases due to variations in search time, keyword settings and software limitations, however we aim to address these limitations in future research.

## Data Availability

The original contributions presented in the study are included in the article/Supplementary material, further inquiries can be directed to the corresponding author.
