# Supplementary material for: Worldwide research trends of pregnancy hypertension in epigenetics field
Source: Front Public Health. 2025 Feb 18;13:1506992. doi: 10.3389/fpubh.2025.1506992 (PMC11876557; doi:10.3389/fpubh.2025.1506992)
Supplement: Supplementary file 1 [file Data_Sheet_1.docx]

**Supplementary Material 1.** Related studies of DNA methylation in HIPE.

| **Gene** | **Expression** | **Source** | **Function** |
| --- | --- | --- | --- |
| STAT5A | Hypermethylated | Placenta and maternal blood | Transcriptiom activation |
| HLA-G | Hypermethylated | Placenta | Maternal lmmune tolerance and immune rejection |
| SERPINB5 | Hypomethylated | Placenta and matemal plasma | Trophoblast Invasion |
| LEP | Hypomethylated | Placenta | Cell homeostasis and metabolism |
| NDRG1 | Hypomethylated | Placenta | Trophoblast invasion |
| BHLHE40 | Hypomethylated | Placenta | Inhibition of trophoblast diferentiation |
| ABCA1 | Hypomethylated | Maternal white blood cell and placenta | Cholesterol transporter in macrophages |
| CRHBP | Hypermethylated | Placenta | Cortisol bioavailability in the placenta |
| GNAS | Hypomethylated | Matermal whiteblood cell and placenta | Diabetes, hypertension and metabolic diseases |
| TAPBP | Hypomethylated | Maternal white blood cell and placenta | Peptide loading in the Histocompatibility complex |
| IGF-1 | Hypermethylated | Placenta | Placentation, trophoblast function, fetal growth |
| DYNLLI | Hypomethylated | Maternal white blood cell and placenta | Phosphate metabolic processing |
| ORPD1 | Hypomethylated | Maternal white blood cell and placenta | Opioid Receptor |
| TIMP3 | Hypomethylated | Placenta | Metalloprotease Inhibitor |
| P2RX4 | Hypomethylated | Placenta | Apoptosis and Inflammation |
| PAPPA2 | Hypomethylated | Placenta | Insuline-like growth factor regulator |
| DLX5 | Hypomethylated | Placenta | Trophoblast proliferation and differentiation |
| SERPINA3 | Hypomethylated | Placenta | Inhibition of inflammation, pathogen degradation and tissue remodeling |
| FN1 | Hypomethylated | Placenta | Cell adhesion, trophoblast proliferation,differentiation and apoptosis |
| PKM2 | Hypomethylated | Placenta | Cellular metabolism |
| COL5A1 | Hypermethylated | Placenta | Extracellular matrix |
| FAM3B | Hypermethylated | Placenta | Cytokme activity |
| INHBA | Hypomethylated | Placenta | Inhibition of trophoblast diferentiation |
| CYP1141 | Hypomethylated | Placenta | Trophoblast autophagy and steroidogenie pathway |
| HSD3B1 | Hypomethylated | Placenta | Steroidogenic pathway |
| CYP19 | Hypomethylated | Placenta | Sterodovenic pathway |
| CRH | Hypomethylated | Placenta | Cortisol bioavailability in the placenla |
| TFPI-2 | Hypomethylated | Placenta | Block im endothelial dysfunction |
| IGF2 | Hypomethylated | Cord blood and placenta | Embryonic development and fetal gmowth |
| GNA12 | Hypomethylated | Placenta and peripheral blood | Blood pressure |
| CAPG | Hypomethylated | Placenta | Macrophage function |
| GLI2 | Hypomethylated | Placenta | Embryo development |
| KRT13 | Hypomethylated | Placenta | Cytoskeleton |
| LP1 | Hypomethylated | Placenta | Lipid metabolism |
| CEBPα | Hypomethylated | Placenta | Transcription stimulation of LEP pmomoter |
| KRT15 | Hypomethylated | Placenta | Cytoskeleton |
| NCAM1 | Hypomethylated | Placenta | Trophoblast-trophoblast interactions and adhesion |
| HSD11B2 | Hypomethylated | Cord blood | Cortisol transmission from the mother to the fetus |
| WNT2 | Hypermethylated | Placenta | Placentation and cell signaling |
| SPESP1 | Hypermethylated | Placenta | Fertilization |
| NOX5 | Hypermethylated | Placenta | Reactive Oxygen Species signaling |
| ALCAM | Hypermethylated | Placenta | Cell Adhesion |
| ADORA2B | Hypermethylated | Placenta | Placenta impaimment and fetal growth restriction |
| SOX7 | Hypermethylated | Placenta | Embryonic development and cell fate |
| CDX1 | Hypermethylated | Placenta | Trophoblast invasion restriction |
| CXCL1 | Hypermethylated | Placenta | Chemokine inducer of angiogenesis |
| CDH11 | Hypermethylated | Placenta | Trophoblast anchoring to the decidua,syncyhiotrophoblast differentiation |
| SH3PXD2A | Hypomethylated | Placenta | Trophoblast invasion and podosome formation |
| SYNE1 | Hypermethylated | Placenta | Nuclear orcanization and structural intecrity |
| AGAP1 | Hypermethylated | Placenta | Cellular development, assembly and function |
| VEGF | Hypomethylated | Pacenta | Anglogenesis |
| RASSF1A | Hypermethylated | Placenta and matemal plasma | Tumor suppressor gene |
| JUNB | Hypomethylated | Placenta | INF signaling pathway |
| PTPRN2 | Hypermethylated | Placenta | Phosphate melabolic processing |
| GATA4 | Hypermethylated | Placenta | Placenta Growth |
| YWHAQ | Hypermethylated | Placenta | Cellular response to reduce oxygen levels |
| TNF | Hypermethylated | Placenta | MMP-9 stimulation, lmmune system activation,cell survival, migration and diferentiation |
| TEAD3 | Hypomethylated | Placenta | Cell homeostasis, Inflammation, Coagulation,complement activation,steroidovenic pathway |

Note:1. The statistical deadline of this table is the date of literature search. 2. The table shows only part of the verified results. 3. In the table, the first column is the gene with DNA methylation difference, the second column is the methylation difference state, the third column is the methylation sequencing organization, and the fourth column is the possible mechanism of the gene causing HIP.

**Supplementary Material 2.** Related studies of miRNA in HIPE.

| **miRNAs** | **Source** | **Expression** | **Target** | **Function** |
| --- | --- | --- | --- | --- |
| miR-18a | Placenta and maternal plasma | downregulated | SMAD2 | Promoting trophoblast migration |
| miR-19b1 | Maternal plasma | downregulated | SMAD | TGFβ-signaling |
| miR-148a | Maternal plasma | upregulated | HLA-G | Immune response |
| miR-582 | Maternal plasma | upregulated | VEGF | Trophoblast invasion and migration |
| miR-152 | Placenta and maternal plasma | upregulated | HLA-G | Immune response |
| miR-300 | Maternal plasma | upregulated | ETS-1 | Trophoblast differentiation |
| miR-106b | Placental and perpheral blood mononudear cells | upregulated | MMIP-2 | Invasion and proliferation |
| miR-126 | Placental and endothelial progenitor cells | downregulated | PIK3R2 | Proliferation, differentiation,migration and vascular sprouting |
| miR 128a | Placental | upregulated | Bax | Apoptosis |
| miR-133 | Placental | upregulated | Rho/ROCKsignaling pathway | Apoptosis |
| miR-136 | MSCs eosomes of peripheralblood and umbilical cord mesenchymal stem cells | upregulated | BCL2/VEGF | Suppress prollferation and promoteapoptosis of MSCs |
| miR-141 | Placental ,peripheral blood | upregulated | CXCL12β/MMP-2/p62/LC3B/ROCK1/RhoA/EG-VEGF | Apoptosis, invasion and vascularizaton |
| miR-141-30 | HUECs | upregulated | Notch2 | Tube fomation, migration, invasionand apoptosis |
| miR-145-5p | Placental and cord blood | downregulated | FLT1/Cyr61 | Trophoblast vability and invasion |
| miR-155 | Placental and maternal plasma | upregulated | eNOS | Invasion and migration |
| miR-16 | Placental | upregulated | Notch2 | Proliferation, migration and invasion angiogenesis |
| miR-17 | Placental | upregulated | ephrin-B2/Eph-B4 receptor | Invasion, proliferation, apoptosis and migration |
| miR-181a-5p | Placental and maternal plasma | upregulated | IGF2BP2 | Trophoblast wability, migration, and invasion |
| miR-196a-5p | Placental | downregulated | SNX16/BHLHE40 | Proliferation, migration and invasion |
| miR-203 | Placental and maternal vessel | upregulated | VEGFA | Cell differentiation |
| miR-205 | Placental and maternal plasma | upregulated | MED1 | Proliferation, migration and invasion |
| miR-20a | Placental | upregulated | FOXA1 | Proliferation, migration and invasior |
| miR-20b | Placental | upregulated | MMP-2/MCL-1 | Prolliferation, migration,apoptosis,invasion,vascular remodeling and growth,tubelike fomation |
| miR-210 | Placental and maternal plasma | upregulated | HIF-1a,/HIF-2α/HSD17B1/E2/PTPN2/KCMF1/THSD7A/NOTCH1 | Capabilities |
| miR-214-5p | Placental | Upregulated | Jagged1/Notch | Proliferation, migration and invasion |
| miR-218 | Placental | upregulated | LASP1 | Invasion |
| miR-2185p | Placental | downregulated | TGFB2 | Invasion and differentiation |
| miR-299 | Placental | upregulated | HDAC2 | Invasion and migration |
| miR-29b | Placental and dMSCs | upregulated | MCL1/MMP-2/VEGFA/ITGB1/HDAC4 | Apoptosis, invasion, angiogenesis,proliferation, migratory and tubule formation |
| miR-30a 3p | Placental | upregulated | IGF-1 | Invasion and apoptosis |
| miR-335-5p | Placental | upregulated | ROS/p53/Sp1 | Migration and EMT |
| miR-342-3p | Placental and maternal plasma | upregulated | ID4/PDGFRA | Cell viabiity, apoptosis and invasion,proliferation, migration, and G1/S phase transition |
| miR-346 | Placental and maternal plasma | upregulated | LRP6 | Proliferation, migration and invasion |
| miR-376c | Placental and maternal plasma | downregulated | ALK5/ALK7/TGF-b/Nodal | Proliferation, migration and invasion |
| miR-378a-5p | Placental | downregulated | Nodal | Cell growth, survival, migration andinvasion |
| miR-454 | Placental | downregulated | Nodal/ALK7/EPHB4 | Proliferation and invasion |
| miR-494 | dMSCs | upregulated | CDK6/OCND1/VEGF | Arrests G1/S transition, migrationand impairs HUVEC capillary formation |
| miR-495 | Cord tissue and dMSCs | upregulated | - | Cell cycle, apoptosis, migration,invasion and tube fomation |
| miR-517 | Placental and maternal plasma | upregulated | sFLT1/ERK/MMP-2 pathway | Prollferative and invasive |
| miR-520g | Maternal serum | upregulated | MMP-2 | Migration and invasion |
| miR-646 | Human peripheral blood-derived endothelial progenitor cells | upregulated | VEGF-A/HIF-1α | Differentiation,migration and angiogenesis |
| miR-93 | Maternal plasma | upregulated | MMP-2 | Inhibits invasion |
| miR-338-3P | Peripheral blood and placental | upregulated | AKT3 | Inhibits proliferation, invasion,metastasis and apoptosis resistance |

Note:1. The statistical deadline of this table is the date of literature search. 2. The table shows only part of the verified results. 3. In the table, the first column is the differentially expressed miRNA, the second column is the detected tissue, the third column is the expression change of the miRNA, the fourth column is the regulatory target gene of miRNA in HIP disease, and the fifth column is the possible mechanism of miRNA causing HIP.

**Supplementary Material 3.** Related studies of lncRNA in HIPE.

| **lncRNAs** | **Source** | **Expression** | **Target** | **Function** |
| --- | --- | --- | --- | --- |
| CAAT1 | placentae | upregulated | E2F1, cyclin D | Decreases proliferation |
| DLX6-AS1 | placentae | upregulated | miR-149–5p | Inhibits proliferation,invasion, angiogenesis,promotes cell apoptosis |
| GAS5 | placentae | upregulated | miR-21 | Inhibits proliferation,migration, invasion |
| INHBA-AS1 | placentae | upregulated | CENPB | Inhibits proliferation,migration and invasion,promotes apoptosis |
| SH3PXD2A-AS1 | placentae | upregulated | SH3PXD2A and CCR7 | Inhibits invasion,migration,proliferation,promotes cell death |
| uc003fir | placentae | upregulated | CCL5 | Promotes proliferation,migration, invasion |
| Linc00473 | placentae | downregulated | LSD1 | Inhibits cell proliferation,migration,invasion,angiogenesis,promotes apoptosis |
| AK002210 | placentae | downregulated | Mir-590-3p | Inhibits proliferation,migration, invasion |
| CRNDE | placentae | downregulated | mir-1277 | Suppresses proliferation,invasion,migration and EMT formation |
| FAM99A | placentae | downregulated | caspase-3,caspase-9,Bax,Wnt /β | Suppresses migration,invasion,increases apoptosis |
| FOXD2-AS1 | placentae | downregulated | MMP2 and MMP9 | Inhibits proliferation,invasion, migration |
| GASAL1 | placentae | downregulated | SRSF1 | Inhibits proliferation,invasion,increases apoptosis rate |
| HIF1A-AS2 | placentae | downregulated | LSD1 and PHLDA1 | Inhibits proliferation,migration, invasion,increases apoptosis |
| SNHG12 | placentae | downregulated | MMP-2 and MMP-9,β-catenin | Inhibits proliferation,migration, invasion |
| SNHG14 | Maternal Blood | downregulated | mir-330-5p | Suppresses proliferation,migration, invasion, EMT |
| MVIH | placentae | downregulated | Jun-Bprotein | trophoblast cell growth,migration and invasion, |
| AGAP2-AS1 | Placental | downregulated | miR-574-5p/JDP2/FOXP1 | proliferation, invasion and apoptosis |
| ATB | Placental | downregulated | - | migratlon, proliferation, and tube fomatlon |
| CCAT1 | Placental | upregulated | D1-P16-CDK4 | cell prollferation and cell cycle |
| DC | Dendritic cells | upregulated | p-STAT3 | invasion |
| EGFR-AS1 | Placental | downregulated | EGFR-JAK/STAT | proliferation |
| H19 | Placental | upregulated | PI3K/AKT /mTOR | cell viability,invasion,autophagy |
| H19 | Exosomes secreted by MSCs | upregulated | microRNA let-7b/FOXO1 | invasion, migration and apoptosis |
| H19 | Placental | downregulated | miR-657 and NOMO1 | proliferation of trophoblast cells observed in EOPE |
| HOTAIR | Placental | upregulated | miR-106a/EZH2 | prolliferation, migration and invasion |
| HOTTIP | Placental | downregulated | RND3 | cell proliferation and cell cycle |
| KCNQ10T1 | Placental | downregulated | miR-146a-3p/CXCL12/CXCR4 | proliferation, invasion and migration |
| Linc00261 | Placental | upregulated | miR-558/TIMP4 | invasion, migration and apoptosis |
| LINC00511 | Placental | downregulated | miR-31-5p/HOXA7/AP2γ | proliferation, invasion and autophagy |
| uc.187 | Placental | upregulated | PCNA/Ki67 and caspase-3/Bcl-2 | proliferation, invasion and apoptosis |
| lncZBTB39 | Placental | upregulated | miR-210/THSD7A | invasion and migration |
| MALAT1 | Placental | downregulated | - | proliferation, migration and invasion,apoptosis |
| MALAT1 | Placental | downregulated | FOS | EMT migration and invasion |
| MALAT1 | Placental | downregulated | miR-206/IGF-1 | migratlon and invasion |
| MALAT1 | Cord tissues and MSCs | downregulated | VEGF/IDO | proliferation, angiogenesis and Immunosuppressrve |
| MEG3 | Placental | downregulated | NF-kB,Caspase-3,and Bax | apoptosis and migration |
| MIR503HG | Placental | upregulated | NF-kB,MMP-2/-9, the snail protein,and E-cadherin | proliferation, invasion,migratlon, apoptosis and cell cycle |
| NR_002794 | Placental | upregulated | - | proliferation, invasion and apoptosis |
| PGK1P2 | Placental | downregulated | miR-330-5p | anglogenesis, glycolsis metabolism and decidualization |
| PVT1 | Placental | downregulated | PI3K/AKT pathway | proliferation, migration and invasion |
| RP11-465L10.10 | Placental | downregulated | MMP-9 | invasion |
| RPAIN | Placental | upregulated | MMP2 ,MMP9 andC1q | proliferation, invasion and apoptosis |
| SNHG5 | Placental | downregulated | miR-26a-5p/N-cadherin | proliferation, migration and invasion |
| SPRY4-IT1 | Placental | upregulated | Wnt/β-catenin pathway | proliferation, migration and apoptosis |
| Storkhead box2 | Placental | upregulated | STOX2 | differentiation and invasion |
| TCL6 | Placental | upregulated | PTEN | cell proliferation and cell cycle |
| TDRG1 | Placental | downregulated | miR-214-5p, Notch | proliferation, migration and invasion |
| TUG1 | Placental | downregulated | miR-204-5p/miR-29b/MCL1/VEGFA/MMP-2/EZH2/RND3/miR-29a-3p | migration, invasion,proliferation, apoptosis and angiogenesis |
| ZEB2-AS1 | Placental | downregulated | miR-149/PGF | proliferation, migration and invasion |

Note:1. The statistical deadline of this table is the date of literature search. 2. The table shows only part of the verified results. 3. The first column of the table shows differentially expressed LNcrnas, the second column shows detected tissues, the third column shows the expression changes of this lncRNA, the fourth column shows the regulatory target genes of lncRNA in HIP diseases, and the fifth column shows the possible mechanism of lncRNA causing HIP.
